# Radiation therapy is an important factor to improve survival in pediatric patients with head and neck rhabdomyosarcoma by enhancing local control: a historical cohort study from a single center

**DOI:** 10.1186/s12887-020-02165-y

**Published:** 2020-05-29

**Authors:** Yuan Wen, Dongsheng Huang, Weiling Zhang, Yi Zhang, Huimin Hu, Jing Li

**Affiliations:** Pediatric Department of Beijing Tongren Hospital, Capital Medical University, 100730, 1# Dong Jiao Min Xiang, Dongcheng District, Beijing, China

**Keywords:** Rhabdomyosarcoma, Pediatric, Radiation therapy, Prognosis, Head and neck, Tumor size

## Abstract

**Background:**

The purpose of this study is to analyze the influence of radiation therapy on survival in a historical cohort of 56 pediatric patients with head and neck rhabdomyosarcoma.

**Methods:**

A historical cohort of 56 pediatric patients with head and neck rhabdomyosarcoma from June 1st, 2013 to June 30th, 2019 was chosen. Clinical data and follow up results were collected including all diagnosis, treatment and prognosis information. Overall survival (OS) and event free survival (EFS) as time-to-event distributions were estimated with Kaplan-Meier method, and univariate analysis was performed with log rank test to detect differences between groups. Multivariate analysis was performed to explore the risk factors for survival with Cox proportional hazard model.

**Results:**

The media follow up time of all 56 patients was 31.8 months (range 3.5–74.6 months). There were 26 events during follow up, including 14 disease progressions and 12 relapses. The estimated 5-year OS of all patients was 69.9%, and the estimated 5-year EFS was 48.8%. Patients with radiation therapy as a component of the initial treatment plan had better 5-year OS and EFS compared with those without radiation therapy (OS 80.3% vs. 49.7%, *p* = 0.003 and EFS 63.9% vs. 21.9%, *p* < 0.001). In patients with events, those who received salvage radiation therapy had better 5-year OS compared with those who didn’t (OS 66.0% vs. 31.2%, *p* = 0.033). On multivariate analysis, tumor size > 5 cm and non-initial radiation therapy were independent risk factors for OS in all patients, non-initial radiation therapy was an independent risk factor for EFS in all patients, and tumor size > 5 cm was an independent risk factor for OS in patients with events.

**Conclusions:**

Radiation therapy as a component of initial treatment can improve the OS and EFS in pediatric head and neck rhabdomyosarcoma patients by enhancing local control, and non-initial radiation therapy is an independent risk factor for OS and EFS. Salvage radiation therapy still can improve OS in patients with disease progression and relapse. Tumor size > 5 cm is an independent risk factor for OS in pediatric HNRMS patients with or without disease progression/relapse.

## Background

Rhabdomyosarcoma (RMS) is the most common childhood soft tissue sarcoma, accounting for about 50% of all patients [1, 2]. It comprises about 4.5% of all childhood cancer with an annual incidence of 4.5 cases per 1 million children and young adults aged under 20 years [3, 4]. This aggressive malignant tumor can develop in any part of the body, and is thought to have a primitive mesenchymal cell origin, with a propensity for striated muscle differentiation [3–5]. The treatment of RMS is a multimodal strategy, referring to the combination of chemotherapy, surgery, and radiation therapy (RT), as well as recent biologically targeted agents [2, 3, 6]. Over the last 3 decades the survival of RMS patients has improved substantially with 5-year OS exceeding 70% [2, 3]. But the prognosis of relapsed and metastatic patients remained poor with 5-year OS about 30% [2, 3, 6]. The prognosis of all RMS is strongly determined by the ability of achieving local control (control of the primary tumor site) [2, 7]. The main pattern of treatment failure including disease progression and relapse is local failure, and maintaining local control is of crucial importance throughout the treatment plan [7, 8]. The two major modalities of local control are surgery and RT, which could be used separately or combined [2, 9].

Head and neck is the most common region of presentation, comprising 40% of all RMS [10, 11]. The primary sites of head and neck rhabdomyosarcoma (HNRMS) include orbit, parameningeal and nonparameningeal nonorbit head and neck. About 50% of HNRMS cases are parameningeal type (unfavorable site), arising in the middle ear/mastoid, nasopharynx/nasal cavity, paranasal sinus, parapharyngeal region, or pterygopalatine/infratemporal fossa [10, 11]. Local control is a significant challenge for HNRMS, especially for parameningeal type [12, 13]. Considering the complicated anatomy of this region, radical surgery would usually cause severe functional and/or cosmetic sequelae, and in most patients there would be gross residual or only a biopsy could be performed [11]. Under this circumstance RT becomes the only appropriate local control method before a second look or delayed primary excision [14].

We found in some of our pediatric HNRMS patients RT was not included as a component of the treatment plan, which was mainly attributed to parental refusal due to different personal considerations. This provided us with the possibility to compare the prognosis of these patients with others. Based on the above we tried to analyze whether RT could improve the survival in pediatric HNRMS patients, and to add evidence to it.

## Methods

### Study design

This is a historical observational cohort study, based on all HNRMS patients diagnosed and treated in our pediatric department from June 1st, 2013 to June 30th, 2019.

### Diagnostic evaluation and risk stratification

The pretreatment diagnostic workup options included head and neck computed tomography (CT) scan and/or magnetic resonance imaging (MRI) with contrast, positron emission tomography-computed tomography (PET-CT) scan, chest CT, radionuclide bone scan, bone marrow aspirates and/or trephine biopsies, cerebrospinal fluid (CSF) test (parameningeal patients). The site, size (widest dimension) and invasiveness of the primary tumor, regional nodal involvement, and metastatic status were determined. Stage was assigned according to the pretreatment staging system of Soft Tissue Sarcoma Committee of Children’s Oncology Group (COG) [15]. (Table 1).

**Table 1:** TNM pretreatment staging system

Stage	Site^a^	T^b^	Size	N^c^	M^d^
1	Nonparameningeal	T1 or T2	Any	Any	M0
2	Parameningeal	T1 or T2	≤5 cm	N0 or Nx	M0
3	Parameningeal	T1 or T2	≤5 cm	N1	M0
			>5 cm	Any	M0
4	Any	T1 or T2	Any	Any	M1

a. For HNRMS favorable site refers to nonparameningeal site (orbit and nonorbit nonparameningeal head and neck); unfavorable site refers to parameningeal site

b. T1, confined to primary site; T2, surrounding tissue invasion

c. Regional nodes N0, not involved; N1, involved; Nx, status unknown

d. M0, no distant metastasis; M1 distant metastasis (includes positive cytology in CSF, pleural, or peritoneal fluid)

Surgical plan was determined based on pretreatment workup results. Excision was attempted on condition of no severe functional and/or cosmetic consequences. Otherwise only biopsy was done. Group was assigned according to intergroup rhabdomyosarcoma study (IRS) surgical-pathologic group system [15]. (Table 2) Pathologic subtype was classified according to the fourth edition of the World Health Organization classification of tumors of soft tissue and bone, which comprise 4 subtypes including embryonal, alveolar, spindle cell/sclerosing and pleomorphic (only seen in adults) subtypes [16].

**Table 2:** IRS surgical-pathologic group system

Group	Definition
I	Localized disease, completely resected
II	Total gross resection, with evidence of regional spread
A	Grossly resected tumor with microscopic residual disease
B	Involved regional nodes completely resected with no microscopic residual disease
C	Involved regional nodes grossly resected with evidence of microscopic residual disease
III	Biopsy only or incomplete resection with gross residual disease
IV	Distant metastatic disease (excludes regional nodes and adjacent organ infiltration)

At last the risk group was assigned with comprehensive consideration of stage, group, and pathologic subtype results, according to the risk group classification of Soft Tissue Sarcoma Committee of Children’s Oncology Group [15]. (Table 3) And chemotherapy was generally guided by the risk group classification.

**Table 3:** COG risk group classification

Risk Group	Histology	Stage	Group
Low	Embryonal	1	I, II, III
	Embryonal	2, 3	I, II
Intermediate	Embryonal	2, 3	III
	Alveolar	1, 2, 3	I, II, III
High	Embryonal or Alveolar	4	IV

### Treatment protocol

The chemotherapy we used for low risk group patients was COG D9602 subgroup B VAC (vincristine, dactinomycin, cyclophosphamide) regimen [17]. For intermediate risk group patients, the COG D9803 standard VAC regimen was used [18]. For high risk group patients, as well as patients with disease progression or disease relapse, based on the standard VAC regimen, an optional combination with anthracyclines, platinum drugs, etoposide or irinotecan was frequently used.

RT was recommended to all patients except for Group I embryonal patients. Generally, according to the recommendation of Soft Tissue Sarcoma Committee of Children’s Oncology Group, the RT dose was 36Gy for Group I alveolar patients, 36Gy or 41.4Gy for Group II patients according to nodal involvement status, 45Gy for Group III orbit patients, and 50.4Gy for other Group III patients. Group IV patients were irradiated as for other groups, including metastatic sites if possible. RT is initiated within 12 weeks after chemotherapy, and radiosensitizing agents were omitted during RT.

Second-look surgery, delayed primary excision, or salvage excision was considered only if no severe functional and/or cosmetic consequences were anticipated.

### Follow up

All patients were closely followed up since diagnosis. Clinical data during and after treatment were recorded. Frequency of off-therapy surveillance was every 3 months for the first year, every 4 months for the second and third year, and once a year for the fourth and fifth year [8]. Clinical physical examination, blood routine and biochemical tests, head and neck CT or MRI with or without contrast, and chest CT or chest X-ray were required for surveillance, and PET-CT was optional to replace all imaging examinations.

Overall survival (OS) was defined as survival from diagnosis to death of any cause. Disease progression (PD) was defined as primary tumor enlargement, and/or new lesions, and/or metastasis during primary treatment course. Disease relapse (RD) was defined as recurrence of RMS in any form after last treatment. Event free survival (EFS) was defined as survival from diagnosis to the first event of PD, RD, second tumor or death of any cause [7, 19, 20]. In this study only the first PD and RD were discussed and analyzed. The patterns of PD and RD included local (primary site), regional (regional lymph node), metastatic, and any combinations.

### Grouping and statistical methods

According to whether RT was included as a component of initial treatment plan, patients were divided into initial RT group (Group IRT) and non-initial RT group (Group NIRT). According to whether RT was included as a component of salvage treatment plan, patients with events, including all PD and RD patients, were divided into salvage RT group (Group SRT) and non-salvage RT group (Group NSRT).

OS and EFS as time-to-event distributions were estimated with Kaplan-Meier method, and survival rates were estimated. Univariate analysis was performed with log rank test to detect differences between groups and Bonferroni adjustment was used to control type I error if more than two groups were compared. Multivariate analysis was performed using Cox proportional hazard model to explore risk factors and adjust confounding factors, and hazard ratios (HR) with 95% confidence intervals (CIs) were calculated. Categorical variables were compared using chi-squared test or Fisher’s exact test between groups. A *p* value < 0.05 was considered statistically significant. Data were analyzed with IBM SPSS Statistics 26.0.

## Results

### Patients’ clinical characteristics

From June 1st, 2013 to June 30th, 2019, 56 pediatric patients were admitted into our pediatric department, who were diagnosed as HNRMS with pathological confirmation. These patients formed our cohort and were diagnosed, stratified, treated and followed up by uniform protocol. The median follow up time was 31.8 (range 3.5–74.6) months for all patients, 37.6 (range 6.1–74.6) months for Group IRT, 20.6 (range 3.5–71.6) months for Group NIRT, 35.7 (range 6.1–74.6) months for Group SRT, and 20.9 (range 4.8–58.4) months for Group NSRT. The specific clinical characteristics of all patients and patients of different groups are showed in Table 4.

**Table 4:** Clinical characteristics of all patients and patients of different groups

	All patients n (%)	All patients			Patients with events		
		IRT n	NIRT n	p	SRT n	NSRT n	p
Total cases	56 (100%)	34	22	–	15	11	–
Gender							
Male	31 (55.4%)	22	9		6	5	
Female	25 (44.6%)	12	13	0.080	9	6	1.000**#**
Age at diagnosis							
≤ 1 y or ≥ 10 ys	17 (30.4%)	10	7		4	4	
>1–9 ys	39 (69.6%)	24	15	0.848	11	7	0.683**#**
Site of origin							
Orbit	22 (39.3%)	14	8		9	1	
Parameningeal	32 (57.1%)	19	13		4	10	
Other head & neck	2 (3.6%)	1	1	0.813*	2	0	0.002*#
Tumor size							
≤ 5 cm	37 (66.1%)	25	12		11	4	
>5 cm	19 (33.9%)	9	10	0.143	4	7	0.109**#**
Histologic subtype							
Embryonal	27 (48.2%)	16	11		6	5	
Alveolar	29 (51.8%)	18	11	0.830	9	6	1.000**#**
Primary tumor invasiveness							
T1	20 (35.7%)	12	8		7	2	
T2	36 (64.3%)	22	14	0.935	8	9	0.217**#**
Regional nodal involvement							
N0	30 (53.6%)	21	9		9	4	
N1	26 (46.4%)	13	12	0.126	6	7	0.428**#**
Metastasis							
M0	49 (87.5%)	32	17		13	9	
M1	7 (12.5%)	2	5	0.099**#**	2	2	1.000**#**
TNM pretreatment stage							
Stage 1	24 (42.9%)	15	9		11	1	
Stage 2	7 (12.5%)	5	2		0	2	
Stage 3	18 (32.1%)	12	6		2	6	
Stage 4	7 (12.5%)	2	5	NA	2	2	NA
Surgical-pathologic group							
Group I	0 (0%)	0	0		0	0	
Group II	8 (14.3%)	5	3		3	0	
Group III	41 (73.2%)	27	14		10	9	
Group IV	7 (12.5%)	2	5	NA	2	2	NA
Risk group							
Low risk	11 (19.6%)	6	5		5	1	
Intermediate risk	38 (67.9%)	26	12		8	8	
High risk	7 (12.5%)	2	5	NA	2	2	NA

IRT: initial RT; NIRT: non-initial RT; SRT: salvage RT; NSRT: non-salvage RT.

*: Tested between parameningeal and nonparameningeal (orbit+other head & neck) groups

#: Fisher’s exact test

NA: not tested because of limited sample size

### Patients’ survival results

There were 26 events observed during follow up, including 14 disease progressions and 12 disease relapses. The pattern of events is showed in Table 5, and the vast majority of events (24/26) belonged to local events (21 local and 3 local+metastatic).

**Table 5:** Pattern of events (disease progression and relapse).*

	Disease Progression (n)	Disease Relapse (n)	Total
Local	12	9	21
Regional	0	1	1
Metastatic	0	1	1
Local+Metastatic	2	1	3
Total	14	12	26

*Only first progression and first relapse were analyzed

**Fig. 1:**
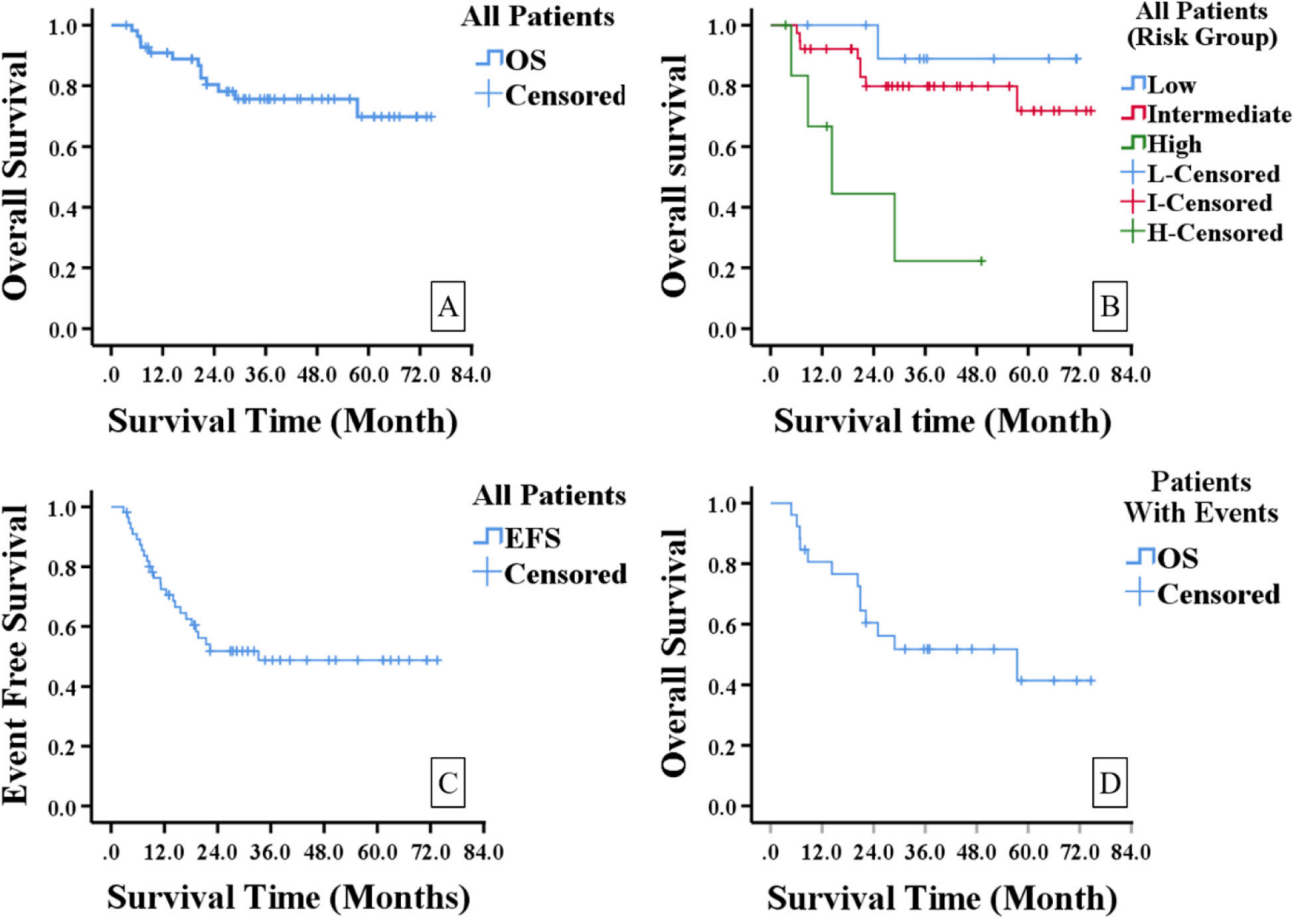
Kaplan-Meier survival curve. A. OS of all patients. B. OS of different risk groups. C. EFS of all patients. D. OS of patients with events (disease progression and relapse)

The estimated 5-year OS of all patients was 69.9%, and 5-year EFS was 48.8%. (Fig. 1a&c) The estimated 5-year OS of low and intermediate risk group was 88.9 and 79.8%, and the 3-year OS for high group was 22.2%, which were statistically different when compared by log rank test (*p* = 0.002). (Fig. 1b) A further pairwise comparison between the 3 risk groups were done with Bonferroni adjustment, and the results showed statistical survival differences between low and high risk groups (*p* = 0.005), as well as intermediate and high risk groups (p = 0.002), but no difference between low and intermediate groups (*p* = 0.345). The estimated 5-year OS for the 26 patients with events (disease progression and relapse) was 41.4%. (Fig. 1d).

### Univariate analysis of risk factors for survival

The previously reported risk factors for survival [15, 19, 21] were compared between groups by univariate analysis with Kaplan-Meier method and log rank test. The results are showed in Table 6. In all patients, the statistically significant risk factors of 5-year OS include tumor size, primary tumor invasiveness, metastasis, surgical-pathologic group, and initial RT; factors of 5-year EFS include tumor size and initial RT. In patients with events (disease progression and relapse), the statistically significant risk factors of 3-year OS include tumor size, primary tumor invasiveness, metastasis, surgical-pathologic group, and salvage RT.

**Table 6:** Univariate analysis of prognostic factors for survival

Risk Factors	All Patients				Patients with events	
	5-y OS	p	5-y EFS	p	3-y OS	p
Age at diagnosis						
≤ 1 y or ≥ 10 ys	80.9%		49.6%		60.0%	
>1–9 ys	66.1%	0.617	48.4%	0.809	48.9%	0.633
Site of origin						
Parameningeal	56.6%		49.0%		39.3	
Non-Parameningeal	81.9%	0.204	46.6%	0.903	65.6	0.094
Tumor size						
≤ 5 cm	83.5%		56.0%		79.4%	
>5 cm	40.3%	**< 0.001**	30.1%	0.035*	10.4%	**< 0.001**
Histologic subtype						
Embryonal	74.6%		51.8%		35.8% (5-y OS)	
Alveolar	65.5%	0.172	46.9%	0.339	36.4% (5-y OS)	0.203
Primary tumor invasiveness						
T1	95%		49.5%		88.9%	
T2	51.4%	**0.013**	50.0%	0.583	29.6%	**0.006**
Regional nodal involvement						
N0	73.6%		51.4%		60.6%	
N1	67.8%	0.330	40.8%	0.498	42.3%	0.481
Metastasis						
M0	75.7%		51.2%		62.0%	
M1	22.2% (3-y OS)	**< 0.001**	25.0% (15-m EFS)	0.097	0.0%	**0.005**
Surgical-pathologic group						
Group II	100%		62.5%		100%	
Group III	66.8%		48.3%		55.6%	
Group IV	22.2% (3-y OS)	**0.001#**	25.0% (15-m EFS)	0.198	0.0%	**0.007#**
Initial RT						
Yes	80.3%		63.9%		35.8%	
No	49.7%	**0.003**	21.9% (20-m EFS)	**< 0.001**	36.4%	0.178
Salvage RT						
Yes	–		–		66.0%	
No	–	–	–	–	31.2%	**0.033**

# Pairwise comparison (Bonferroni adjustment: *p* < 0.0167 is considered statistically significant)

5-year OS of all patients: Group II vs Group III (*p* = 0.098), Group II vs Group IV (p = 0.003), Group III vs Group IV (p = 0.003)

3-year OS of patients with events (disease progress and relapse): Group II vs Group III (*p* = 0.127), Group II vs Group IV (*p* = 0.018), Group III vs Group IV (*p* = 0.014)

* Breslow test results

**Fig. 2:**
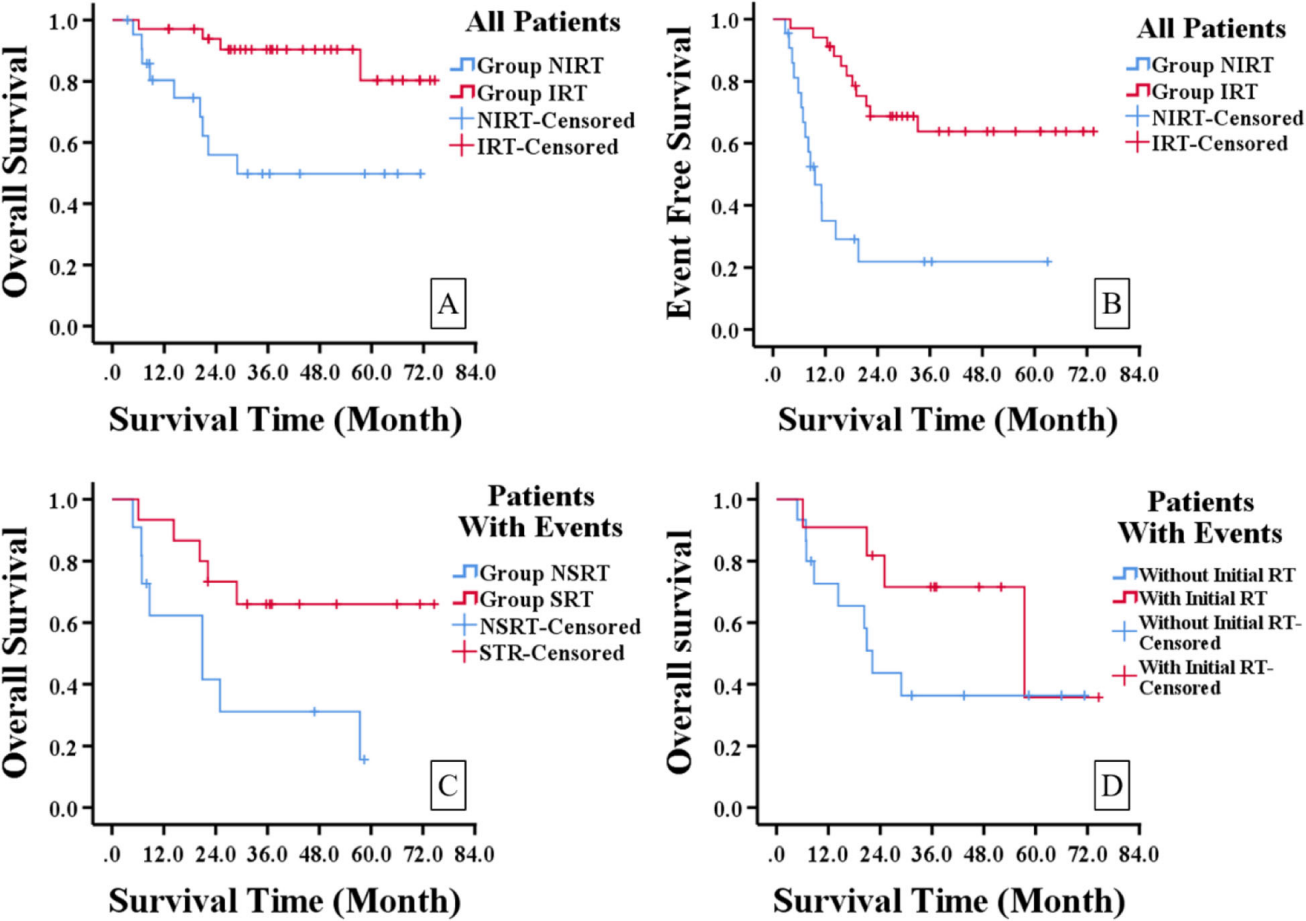
Kaplan-Meier survival curve of patients with or without RT. A. OS of Group IRT and Group NIRT (*p* = 0.003). B. EFS of Group IRT and NIRT (*p* < 0.001). C OS of Group SRT and NSRT (*p* = 0.033). D. OS of patients with events with or without initial RT (*p* = 0.178)

By univariate analysis we noticed that patients treated with initial RT had better OS and EFS than those without. And in patients with events those treated with salvage RT had better OS, but the influence of initial RT showed no statistical significance in these patients. (Fig. 2.)

### Multivariate analysis of risk factors for survival

Cox proportional hazard model was used for multivariate analysis, and all statistically significant variables were included in the model, in order to adjust confounding factors. And considering the collinearity between variable metastasis and variable surgical-pathologic group (patients with metastasis belongs to surgical-pathologic group IV, which means there is an information overlap, and the two variables should not be in the Cox model at the same time), which was confirmed by collinearity diagnostics with condition index 19.33,variance proportion of metastasis 0.69 and surgical-pathologic group 0.93, variable metastasis was excluded from the model. The results were expressed as HR with 95% CIs for each variable, and a variable with *p* value < 0.05 was considered to be an independent prognostic factor. The multivariable analysis results are showed in Table 7. Tumor size > 5 cm and non-initial RT were independent risk factors for OS in all patients, non-initial RT was an independent risk factor for EFS in all patients, and tumor size > 5 cm was an independent risk factor for OS in patients with events.

**Table 7:** Multivariate analysis results of Cox proportional model

Risk Factors	OS (All Patients)			EFS (All Patients)			OS (Patients with Events)		
	HR	95% CIs	p	HR	95% CIs	p	HR	95% CIs	p
Tumor size	5.06	1.17–21.93	**0.030**	1.92	0.86–4.27	0.110	5.61	1.12–28.06	**0.036**
Primary tumor invasiveness	4.59	0.51–41.13	0.173	–	–	–	5.96	0.62–54.59	0.114
Surgical-pathologic group	1.26	0.33–4.84	0.741	–	–	–	1.50	0.31–7.40	0.617
Initial RT	4.66	1.33–16.36	**0.016**	4.69	2.08–10.55	**< 0.001**	–	–	–
Salvage RT	–	–	–	–	–	–	2.06	0.54–7.79	0.289

## Discussion

Most of our HNRMS patients belonged to parameningeal type/unfavorable site (57.1%), Group III/gross residual (73.2%) and intermediate risk group (67.9%). These distribution features were consistent with current published results [22]. Thus, the local control would be more dependent on RT because of the limited chances for a complete primary resection considering the complicated anatomy and functional/ cosmetic consequences [11, 14]. Baseline characteristics (Table 4.) of Group IRT and Group NIRT, as well as Group SRT and Group NSRT were generally comparable, which provided the foundation for further comparisons between groups.

In our cohort during the follow up there were 26 events, of which 24 were local events. This result was consisted with other reports, that the most common treatment failure (including disease progression and relapse) was local failure, and maintaining local control would benefit prognosis substantially [7, 8, 22].

The COG risk group classification is an effective risk stratification strategy, which can evaluate patients’ risk factors comprehensively, guide chemotherapy choice, and predict outcome [2, 3, 15]. Our survival results supported the effectiveness of this risk group classification in predicting outcome, but a pairwise comparison of survival between low and intermediate risk group didn’t reach statistical significance (*p* = 0.345). This may be explained by RT as a confounding factor undermining the survival difference between the two groups, or our limited sample size to detect it. Though we couldn’t prove either of them based on our current cohort, we still can see the trend of survival difference on the Kaplan Meier survival curve (Fig. 1b).

In univariate analysis process we tested some widely accepted prognostic factors in our cohort, and some reached statistical significance. Besides RT, which will be discussed later, we found that statistically significant prognostic factors for OS in all patients and patients with events were very consistent, including tumor size, primary tumor invasiveness, metastasis, and surgical-pathologic group. And besides RT, tumor size is the only statistically significant prognostic factor for EFS. Tumor size is an important prognostic factor for survival in HNRMS, patients with smaller tumor (≤5 cm) have better survival compared with patients with larger tumor (>5 cm) [7, 23]. Tumor size > 5 cm has been widely reported as a risk factor for survival especially in refractory patients (patients with disease progression) and recurrent patients (patients with disease relapse) [24–27]. These results are consistent with our result that tumor size could predict OS in patients with or without events. And tumor size was the strongest predictor of local failure [28], which is consistent with our results that tumor size could predict EFS over other factors.

Our univariate analysis results showed that initial RT was a statistical prognostic factor for both OS and EFS in all patients, but it was not for OS in patients with events. This might indicate that initial RT could help to improve OS by enhancing local control/ preventing local events, but once events occurred, initial RT would become a low-weight prognostic factor. We speculated that after events, salvage RT took the place of initial RT and became a high-weight prognostic factor, and this speculation was supported by our results that salvage RT was a statistically significant prognostic factor for OS in patients with events. It’s reported that adequate local therapy is an important factor for survival after relapse [29], RT can further improve OS in relapsed patients undergone a repeat surgery [30], and local treatment such as RT and repeat surgery should be systemically considered even in previously irradiated patients [31]. The cure of parameningeal RMS is unlikely without RT [11]. In conclusion, our results showed that whether as a component of initial or salvage treatment plan, RT could improve patients’ OS.

In order to adjust confounding factors, further multivariate analyses were performed using Cox proportional hazard model. First, tumor size was proved to be an independent prognostic factor for OS in patients with or without events, which confirmed its important influence on patients’ outcome. Second, initial RT was proved to be an independent prognostic factor for OS and EFS, which confirmed it as a very important modality to improve OS by enhancing local control. Third, salvage RT was not an independent prognostic factor for OS in patients with events. In our cohort patients with events (disease progression and relapse) generally refers to refractory and recurrent patients. These patients’ survival is affected by many factors and some of them may be higher-weight prognostic factors, such as tumor size, primary site, regional nodal involvement, metastasis, repeat surgery, previous chemo and RT plan, multi-relapse, etc. [3, 19, 27, 29, 32]. All these factors make salvage RT unlikely to be an independent prognostic factor.

Over the last 3 decades with the continuous efforts made by large cooperative groups, such as the COG Soft Tissue Sarcoma Committee in North America, the European pediatrics Soft Tissue Sarcoma Study Group (EpSSG), etc., the current 5-year OS for pediatric RMS patients exceeds 70% [2, 3]. For patients with poor prognosis, treatment failure is mainly due to local failure, which refers to primary tumor progression or recurrence, including the combination of local failure with regional nodal failure, and/or metastasis. The key to improve prognosis is to maintain local control. The first thing to cure RMS is the eradication of the primary tumor, which is realized by surgery and/or RT, then at the same time chemotherapy can eradicate micro residual or disseminated tumor cells [2]. It’s reported that patients without RT as a component of the treatment plan have a poor prognosis [33], and in HNRMS patients if the primary tumor is unresectable, RT and chemotherapy are the mainstay of initial treatment [11]. Here we cannot overemphasize the importance of RT in treating pediatric HNRMS patients, and omitting RT may lead to poor prognosis.

Despite the benefit of RT, about 40% (22/56) of our patients’ parents initially rejected the adoption of RT for their children, which are generally due to two reasons: one is the concern about long-term morbidity related with RT such as orbital hypoplasia, eye problems, and pituitary dysfunction, etc., the other is the fact that some kids show very good/complete response to initial chemo regiments, which enhanced parents’ confidence that chemotherapy is reliable and capable of cure. Regarding the two situations, we may consider introducing them less toxic RT modalities, such as proton radiotherapy, brachytherapy, etc., as well as adequately explaining the necessity of RT, the risk of refusing it, and the limited predictive value of initial response to chemotherapy, to ease their concern and enhance their confidence for RT.

### Limitations

This is a single-center historical cohort study with a small sample size, but our uniform diagnostic and therapeutic protocol could also be a strength. Our hospital is a tertiary center with domestically high-ranking ophthalmology and otorhinolaryngology head & neck surgery department, also the fact that we didn’t identify surgical-pathologic group I patients may indicate a selection bias. These factors may limit the generalizability of this study. We can’t acquire the PAX-FOXO1 fusion gene status in nearly half of our alveolar patients, in order not to cause false interpretations it was not analyzed in this study. But PAX-FOXO1 fusion gene status is absolutely a very important prognostic factor and is widely reported [2, 19, 20], not being able to analyze it could be a flaw of this study.

## Conclusions

In conclusion, RT as a component of initial treatment can improve the OS and EFS in pediatric HNRMS patients by enhancing local control, and non-initial RT is an independent risk factor for OS and EFS. Salvage RT still can improve OS in patients with disease progression and relapse. Tumor size > 5 cm is an independent risk factor for OS in pediatric HNRMS patients with or without disease progression/relapse.

## Data Availability

Not applicable.

## Abbreviations

OS: Overall Survival
EFS: Event Free Survival
RMS: Rhabdomyosarcoma
RT: Radiation Therapy
HNRMS: Head and Neck Rhabdomyosarcoma
CT: Computed Tomography
PET-CT: Positron Emission Tomography-Computed Tomography
CSF: Cerebrospinal Fluid
COG: Children’s Oncology Group
IRS: Intergroup Rhabdomyosarcoma Study
VAC: Vincristine, Dactinomycin, Cyclophosphamide.
PD: Disease Progression
RD: Disease Relapse
HR: Hazard Ratio
CI: Confidence Interval
Group IRT: Initial Radiation Therapy Group
Group NIRT: Non-initial Radiation Therapy Group
Group SRT: Salvage Radiation Therapy Group
Group NSRT: Non-salvage Radiation Therapy Group
